# Vaccine-Induced Boosting of Influenza Virus-Specific CD4 T Cells in Younger and Aged Humans

**DOI:** 10.1371/journal.pone.0077164

**Published:** 2013-10-14

**Authors:** Douglas V. Dolfi, Kathleen D. Mansfield, Raj K. Kurupati, Senthil Kannan, Susan A. Doyle, Hildegund C. J. Ertl, Kenneth E. Schmader, E. John Wherry

**Affiliations:** 1 Institute for Immunology, Department of Microbiology, University of Pennsylvania Perelman School of Medicine, Philadelphia, Pennsylvania, United States of America; 2 The Wistar Institute, Philadelphia, Pennsylvania, United States of America; 3 Division of Geriatrics, Department of Medicine, Duke University Medical Center, and Geriatric Research Education and Clinical Center, Durham VA Medical Center, Durham, North Carolina, United States of America; 4 Biomedical Graduate Group, University of Pennsylvania, Philadelphia, Pennsylvania, United States of America; Leiden University Medical Center, Netherlands

## Abstract

Current yearly influenza virus vaccines induce strain-specific neutralizing antibody (NAb) responses providing protective immunity to closely matched viruses. However, these vaccines are often poorly effective in high-risk groups such as the elderly and challenges exist in predicting yearly or emerging pandemic influenza virus strains to include in the vaccines. Thus, there has been considerable emphasis on understanding broadly protective immunological mechanisms for influenza virus. Recent studies have implicated memory CD4 T cells in heterotypic immunity in animal models and in human challenge studies. Here we examined how influenza virus vaccination boosted CD4 T cell responses in younger versus aged humans. Our results demonstrate that while the magnitude of the vaccine-induced CD4 T cell response and number of subjects responding on day 7 did not differ between younger and aged subjects, fewer aged subjects had peak responses on day 14. While CD4 T cell responses were inefficiently boosted against NA, both HA and especially nucleocaspid protein- and matrix-(NP+M) specific responses were robustly boosted. Pre-existing CD4 T cell responses were associated with more robust responses to influenza virus NP+M, but not H1 or H3. Finally pre-existing strain-specific NAb decreased the boosting of CD4 T cell responses. Thus, accumulation of pre-existing influenza virus-specific immunity in the form of NAb and cross-reactive T cells to conserved virus proteins (e.g. NP and M) over a lifetime of exposure to infection and vaccination may influence vaccine-induced CD4 T cell responses in the aged.

## Introduction

Current influenza virus vaccines can induce NAb and protective immunity in many subjects. However, these vaccines are poorly effective in the elderly with vaccine effectiveness (VE) against Influenza A (H3N2) of only 9% in individuals 65 and older for the 2012-2013 season [1]. Even though the 2012-2013 vaccine was designed to elicit neutralizing antibodies to the correct circulating strains (i.e. lack of VE was not due to strain mismatch), the vaccine performed poorly, highlighting the need for understanding more broadly protective immune mechanisms for influenza virus. Furthermore, as VE is an estimate based only on hospitalizations or doctor visits, many more individuals, particularly the elderly, may not be adequately protected during a severe epidemic season. Thus, a major goal is to develop vaccines that elicit broad, heterosubtypic protective responses against influenza virus infection. While promising ideas are emerging including the role of memory CD4 T cells, the impact of a lifetime of recurrent exposure to influenza viruses and vaccination on the ability to elicit broadly protective immunity through vaccination remains poorly understood.

There has been considerable recent interest in influenza virus-specific CD4 T cells as potential targets for heterosubtypic immunity [2-4]. In animal models Th1-like memory CD4 T cells can provide robust heterotypic immunity [5,6]. Moreover, recent human challenge studies suggest that CD4 T cell responses correlate well with outcome of infection [7] and nearly all individuals have CD4 T cells specific for influenza viruses [8]. Recent studies in young subjects indicate a substantial cross-reactivity of CD4 T cell responses for different strains of influenza virus [9], consistent with better sequence conservation outside of NAb determinants. In addition, expansion of CD4 T cell responses following vaccination correlates with NAb responses in young subjects [10,11] suggesting that vaccine-mediated boosting of CD4 T cell responses may be important not only for generating Th1-like memory that can be directly protective [5,12], but also for generating CD4 T cells that can provide help for other components of the immune response.

CD4 T cells become less functional in aged subjects [8,13]. These data are consistent with the observations that aged individuals have decreased trivalent inactivated influenza vaccine (TIV) responsiveness for both antibody and CD4 T cells [14]. While there is a general increase in memory CD4 T cells at the expense of naïve CD4 T cells in aged subjects [15], the number of circulating influenza-specific CD4 T cells does not seem to differ [16]. This observation is interesting considering that the history of exposure to influenza virus infection and vaccination in the elderly might be expected to lead to an accumulation of increased numbers of virus-specific memory CD4 T cells [17]. However, a number of age-related changes in CD4 T cells have been described including defective apoptosis of CD4 T cells [18,19] and decreased cytokine production and expansion [20,21] that may influence the behavior of antigen-specific CD4 T cells in response to influenza virus vaccination in humans. Despite the potential importance of influenza virus-specific CD4 T cell responses as a component of broadly protective immunity in the elderly, the impact of influenza virus-vaccination on these cells later in life remains poorly understood.

Here we examine CD4 T cell responses to TIV in aged and younger humans. Although the magnitude of CD4 T cell responses to TIV were similar in younger and aged subjects, younger individuals had bimodal peaks of vaccine-induced CD4 T cell boosting on days 7 and 14, whereas aged individuals showed fewer peak responses on day 14 compared to day 7. Both pre-existing CD4 T cells specific for influenza virus and pre-existing anti-influenza NAb appeared to impact the magnitude of the CD4 T cell boost following TIV. Pre-existing CD4 T cell responses to internal proteins NP and M were effectively boosted by TIV, whereas responses of H1- or H3-specific CD4 T cells, as well as responses to NA, were not different in subjects with or without pre-existing HA or NA-specific CD4 T cells. Finally, the presence of NAb to HA at the time of vaccination negatively impacted the magnitude of the CD4 T cell boost to HA and NP+M proteins. These data have important implications for vaccine formulation and our understanding of how TIV stimulates cellular immunity in younger versus aged humans.

## Materials and Methods

### Human Subjects

Study subjects were recruited from the Durham, Chapel Hill and Raleigh, NC communities, consented in writing and enrolled at the Duke Geriatric Evaluation and Treatment Clinic at Duke University Medical Center between November 2011 and April 2012 in accordance with and approved by the institutional review boards of both Duke University and the University of Pennsylvania. Subjects were defined as young if they were between 30 and 40 years of age, or aged if they were 65 years of age or older. All subjects were free from other ailments and not currently being treated for other conditions that would impact their pacticipation or the results of these studies. 15 young and 30 aged subjects were recruited and given the FDA recommended TIV for the 2011-2012 season (Fluarix, GlaxoSmithKline Biologicals, A/California/7/09 H1N1, A/Perth/16/2009 H3N2, B/Brisbane/60/2008) on day 0 of the study (Table **1**). PBMCs were isolated from subjects on days 0, 7, 10, 14, 28, and 60 after TIV.

### Peptide and superantigen stimulation for intracellular cytokine production

PBMCs were isolated from peripheral blood using Ficoll-Paque PLUS (GE Healthcare). Directly isolated PBMCs were washed in prewarmed RPMI 1640 containing 10% FCS and rested overnight at 37°C before stimulation. Cells were then stimulated with peptide pools (0.5µg/mL) or superantigen Staphylococcal Enterotoxin F (SEF) (1.0 µg/mL) in the presence of brefeldin A for 5hrs at 37°C before staining. Peptide pools were composed of overlapping 13- to 17-mers with 11 or 12 amino acid overlap. The following reagents were obtained through the NIH Biodefense and Emerging Infections Research Resources Repository, NIAID, NIH: Peptide Array, Influenza Virus A/California/07/2009 (H1N1) Hemagglutinin Protein, NR-1924 (139 peptides) and Neuraminidase Protein, NR-19248 (115 peptides), Influenza Virus A/New York/384/05 (H3N2) Hemagglutinin (HA) Protein, NR-2603 (94 peptides) and Neuraminidase Protein, NR-2608 (78 peptides), Influenza Virus A/New York/384/03 (H1N1) Nucleocaspid Protein, NR-2611 (82 peptides), and Matrix Protein 1, NR-2613 (41 peptides).

### Flow Cytometry

Immediately after peptide or SEF stimulation PBMCs were stained for surface markers and intracellular cytokines. The following antibody conjugations were used: Aqua Live/Dead, CD4 PE-Cy5.5 (Invitrogen), CD14 APC-H7, CD16 APC-H7, CD19 APC-H7 (BD Biosciences), IFN-γ Alexa Fluor 700, IL-2 Brilliant Violet 421, PD-1 PE, TNF-α PE-Cy7, CD27 PerCP-Cy5.5 (Biolegend), CD8 eFluor 650NC, CD45RA eFluor 605NC (eBioscience), CD244 PE-Cy5 (Beckman-Coulter), LAG-3 Biotin (Enzo Life Sciences), CD3 Qdot 585, CD57 Qdot 565, CD160 [22] custom conjugated to FITC. Intracellular cytokines were stained using the BD Cytofix/Cytoperm Kit (BD Biosciences). Samples were acquired on a BD LSR II and analyzed using Flow Jo software (Tree Star). Fluorescence minus one (FMO) controls were performed in initial studies to define positive versus negative staining and determine where marker cutoffs should be set. Gates for cytokine production were determined using no stimulation control performed on each sample. Influenza virus-specific responses presented have had this no stimulation control value subtracted out.

### Micro-Neutralization Assay

Influenza virus-specific micro-neutralization assay was performed on heat-inactivated human sera serially diluted (1:10 to 1:5120) in 96 well plates. The two influenza strains, Influenza A/H1N1/2009/California and Influenza A/H3N2/2009/ Perth at 50 TCID50 was added to serum samples and incubated for 1 hr at 37°C. Serum-virus mixtures were added to Madin Darby canine kidney (MDCK) cells and further incubated for 2 hrs at 37°C with 5% CO2. The plates were washed and MEM containing Tosyl phenylalanyl chloromethyl ketone-modified trypsin added to each well. After incubating for 3 days, plates were scored for cytopathic effect.

### ELISA

To measure H1N1/California and H3N2/Perth- specific antibody isotypes, wells of Nunc Maxisorp^TM^ plate were coated with each virus or IgA1, IgG and IgM (Athens Research & Technology, Inc., Georgia, USA) in bicarbonate buffer overnight at 4°C. Heat-inactivated sera were diluted to 1/250 and added to the plate for 2h at room temperature. Antibodies were detected with alkaline phosphatase conjugated mouse anti-human IgA1 at 1:1000, IgG at 1:3000 and IgM at1:1000 (SouthernBiotech, Alabama, USA) and alkaline phosphatase substrate containing pNPP tablets (Sigma Aldrich, Missouri, USA) in DEA buffer. 

### Statistics

Statistical analysis was done using Excel (Microsoft) and Prism software (GraphPad). Non-parametric Mann-Whitney test, Spearman Correlation, Fisher’s Exact test, and ANOVA were performed as indicated in figure legends.

## Results

### Human CD4 T cells expand and differentiate in vivo in response to TIV

**Figure 1:**
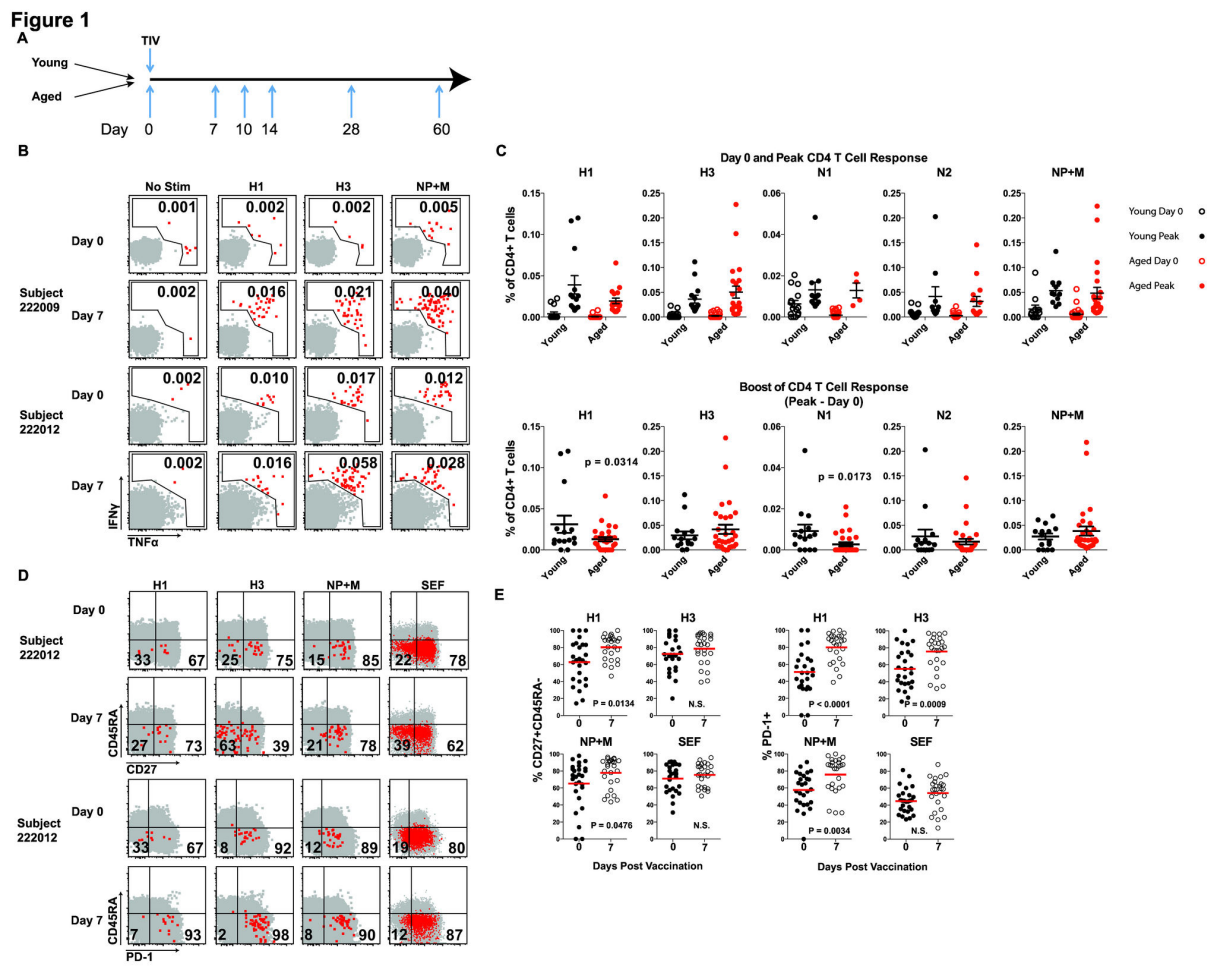
Tracking CD4 T cell responses to TIV in young and aged subjects. (**A**) Vaccine response kinetics were examined at the indicated time points time points. (**B**) For CD4 T cell responses, subjects were monitored by intracellular cytokine staining (ICS) for the production of IFNγ and/or TNFα following stimulation with separate peptide pools for influenza virus proteins. Representative flow cytometry plots show examples of gating from two subjects on day 0 and day 7. Subject 222009 is an example with day 0 responses near background levels while subject 222012 has multiple day 0 responses above background. Both subjects have positive day 7 responses. Numbers in upper right corner indicate the percent of CD4 T cells making either cytokine. (**C**) Combined data in the top panel summarize responses to each peptide pool for day 0 (open circles) or the peak (day 7 or day 14; see Figure 2 below) response (closed circles). Values have had the no stimulation background (as shown in **B**) subtracted from the peptide pool stimulation. The bottom panel displays the same data with day 0 responses subtracted from the peak responses for each individual to quantify the vaccine-induced boost or change from day 0 for each response in each subject. (**D**) Phenotypic changes in total (grey) or responding CD4 T cells (producing IFNγ and/or TNFα following peptide pool stimulation, red) were examined. Representative flow cytometry plots of CD27 and CD45RA expression or PD-1 expression. Numbers indicate percent of cytokine producing (red) CD4 T cells in each quadrant. The same subject 222012 is show in all plots to illustrate differences in responding populations with a given individual. (**E**) Pooled data summarize changes in expression of CD27+CD45RA- or PD-1+ influenza virus-specific CD4 T cells from all subjects (combined data from young and aged) on days 0 and day 7 after TIV. Statistical significance was assessed using Mann-Whitney test for non-parametric data.

To examine the detailed kinetics of CD4 T cell boosting by influenza virus vaccination, we recruited younger and aged individuals, and monitored virus-specific CD4 T cell responses in peripheral blood on days 0, 7, 10, 14, 28, and 60 after vaccination (Figure **1A**). Younger subjects (n=15) ranged in age from 30-40, while aged individuals (n=30) were between 65-87 years old (Table **1**). Influenza virus-specific T cell responses were examined using stimulation with overlapping peptides for HA, NA, NP, and M followed by intracellular cytokine staining for IFNγ and TNFα as described [23]. The limit of detection for this assay was 0.005% of CD4 T cells (Figure **S1**), on par with other highly sensitive assays such as ELISpot. Gating of cytokine responsive influenza virus-specific CD4 T cells was based on no stimulation control condition for each sample (Figure **S2**). The no stimulation control gate was set at or below 0.005% of CD4+ T cells with a mean of 0.0037% and median of 0.0022% (minimum 0.0 and maximum 0.0585% for all subjects and responses). Positive responses ranged from ~0.01-0.25% of CD4 T cells, similar what other groups have shown [7,8,24]. Positive responses were defined as a two-fold increase over background (no stimulation control) and greater than 0.005% of CD4 T cells. The background for each subject was then subtracted from peptide or positive control to determine the magnitude of the antigen-specific response. Vaccine-mediated boosting of CD4 T cells was determined by an increase in cells producing IFNγ and/or TNFα compared to the response at day 0. Representative CD4 T cell responses in Figure **1B** illustrate pre-existing day 0 responses and vaccine-mediated responses on day 7-post vaccination. Day 0 and peak responses to each peptide are shown as background-subtracted responses (Figure **1C** top). The bottom panels in Figure **1C** show the boost of the CD4 T cell response in each subject by quantifying the peak magnitude following vaccination minus the pre-existing day 0 response. TIV-elicited CD4 T cell responses were detected to viral HA proteins from both influenza A virus vaccine strains (H1 14 of 16 younger and 22 of 30 aged and H3 15 of 16 younger and 28 of 30 aged) as well as an overlapping peptide pool for NP and M (12 of 16 young and 21 of 30 aged) protein (Figure **1B and C**). The younger subjects had a slightly better boost of H1 responses compared to the aged subjects. Vaccine-induced CD4 T cell responses to NA were of lower magnitude and/or less common (11 of 16 young and 8 of 30 aged) compared to HA and NP+M (Figure **1C**) and we therefore focused most attention on HA and NP+M for subsequent analyses. CD4 T cells specific for influenza virus are typically mostly memory phenotype [25]. Here, most influenza virus-specific CD4 T cells in both young and aged subjects expressed CD27 and PD-1 and were low for CD45RA (Figure **1D and E**). An increased percentage of virus-specific CD4 T cells at day 7-post vaccination became CD27+CD45RA- and PD-1+ (Figure **1D and E**), consistent with an increase in activation following vaccination. In addition to an increased percentage of PD-1+ cells, more PD-1 was expressed per cell (higher MFI) on day 7-post vaccination (Figure **1E and S3**). No statistical difference in these phenotypic markers before and after vaccination was detected on CD4 T cells responding to SEF stimulation, suggesting that the changes observed for influenza virus-specific CD4 T cells were occurring in an antigen-specific manner due to TIV. These data demonstrate robust CD4 T cell responses to influenza virus vaccine including both a numerical increase and elevated expression of cellular activation markers. These phenotypic changes occurred for NP+M-responding CD4 T cells from vaccinated subjects in addition to CD4 T cells responding to HA suggesting significant responses to these proteins presumably due to NP and/or M contained in the vaccine along with HA and NA [2,26,27]. As both HA and NP+M directed CD4 T cell responses were prominent after TIV, we examined these responses in more detail.

**Table 1:** Subject Enrollment and Demographics.

**Subject #**	**Date enrolled**	**age**	**male**	**female**	**white**	**black**	**other**
111-006	01/04/16	30	1			1	
111-013	02/07/16	30	1		1		
111-001	11/29/15	31		1	1		
111-004	12/06/15	31		1	1		
111-008	01/18/16	31		1			1
111-003	12/06/15	37	1		1		
111-005	12/06/15	37	1		1		
111-009	01/18/16	38	1			1	
111-016	02/22/16	38	1			1	
111-010	01/24/16	39		1		1	
111-011	01/31/16	39		1		1	
111-012	01/31/16	39		1	1		
111-014	02/15/16	39		1	1		
111-015	02/21/16	39		1	1		
111-002	12/06/15	40		1	1		
222-013	03/06/16	65	1		1		
222-016	03/07/16	66		1	1		
222-019	03/13/16	67		1	1		
222-020	03/20/16	67		1	1		
222-028	05/08/16	68		1	1		
222-003	01/18/16	69		1	1		
222-024	04/10/16	70		1		1	
222-002	01/04/16	71		1	1		
222-023	04/10/16	71	1		1		
222-027	04/25/16	71	1				1
222-030	05/08/16	71	1		1		
222-011	02/22/16	73		1	1		
**222-014**	**03/06/16**	**73**		**1**	**1**	**withdrawn**	
222-021	03/20/16	73		1	1		
222-001	11/29/15	74		1	1		
222-017	03/07/16	74		1		1	
222-018	03/13/16	74	1		1		
222-004	01/24/16	76	1		1		
222-009	02/07/16	76		1	1		
222-010	02/15/16	76		1	1		
222-012	02/28/16	76	1		1		
**222-015**	**03/06/16**	**76**	**1**		**1**	**withdrawn**	
222-006	01/31/16	77		1	1		
222-025	04/24/16	78		1			1
222-007	01/31/16	79		1	1		
222-029	05/08/16	80		1	1		
222-026	04/24/16	83	1		1		
222-008	01/31/16	84	1		1		
222-022	04/04/16	85		1	1		
222-005	01/31/16	87		1	1		
**111-007**	**01/18/16**					**withdrawn**	

### Differential influenza virus vaccine CD4 T cell boosting kinetics in young and aged

**Figure 2:**
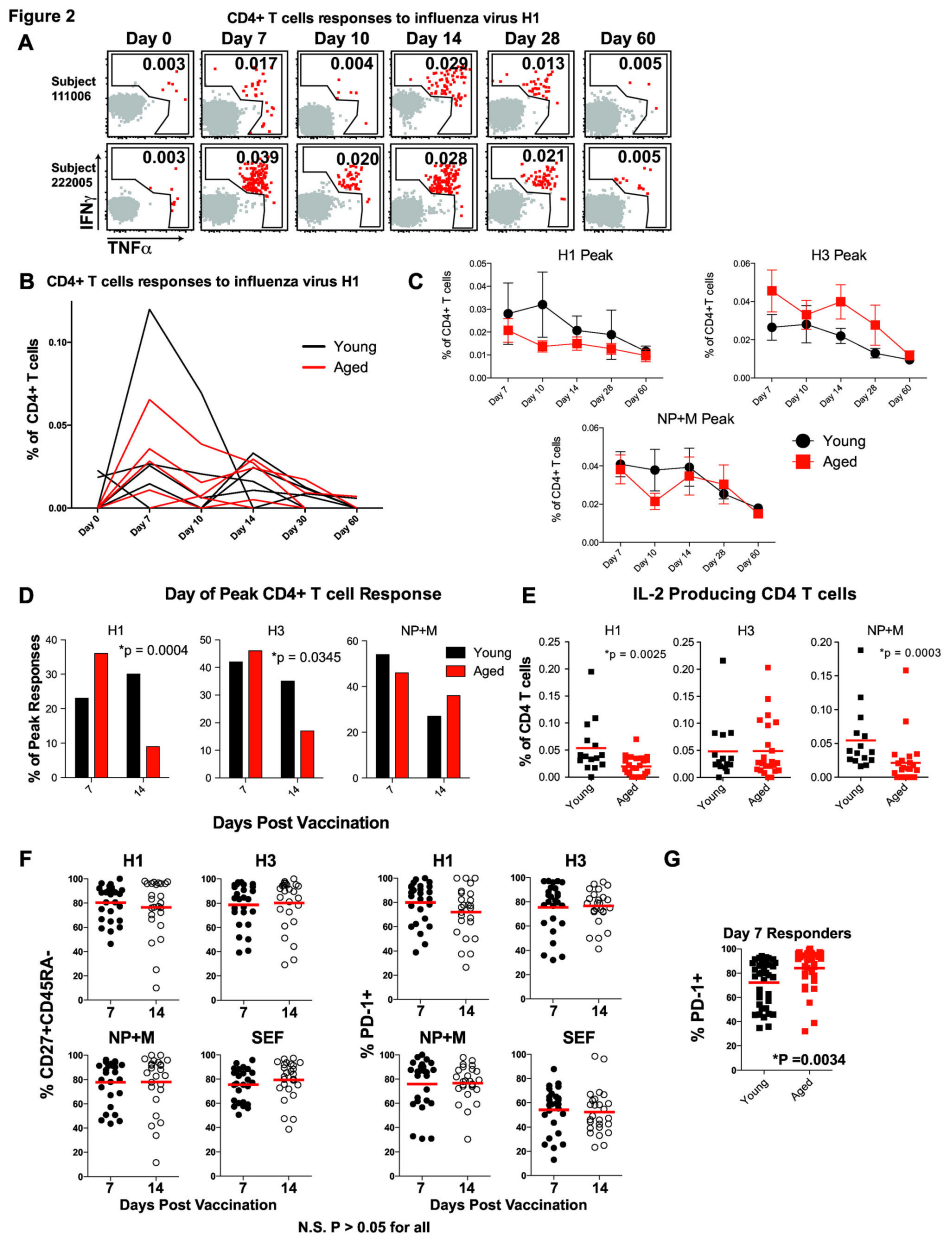
CD4 T cell response kinetics after TIV. (**A** and **B**) Vaccine-induced responses were defined as an increase in cytokine production compared to the d0 response and were examined over time for each subject. Representative flow cytometry plots (**A**) and kinetic graphs (**B**) show changes in cytokine production over time after vaccination. Examples of responses to H1 from young and aged are shown illustrating gating and changing percent of responding CD4 T cells over time (**A**). The numbers in **A** indicate the percent of CD4 T cells making IFNγ and/or TNFα. Line graphs for multiple subjects demonstrate the range and kinetics for multiple young (black) and aged (red) subjects (**B**). Summarized data for young (black) and aged (red) show the mean and standard error for responses to each protein over time (**C**). The number of subjects peaking on day 7 versus day 14 was compared between young (black bars) and aged (red bars) subjects for responses to H1, H3, or NP+M (D). Statistical testing was performed using the Fisher’s exact Chi-squared. Summary data for IL-2 production to each peptide pool is shown for young (black) and aged (red) (Mann-Whitney) (**E**). Differentiation phenotype (CD27+CD45RA- on left or PD-1+ on right) of influenza virus-specific CD4 T cells peaking on day 7 or day 14 post-TIV for individual influenza proteins (**F**). For this analysis young and aged subjects were combined to test whether CD4 T cell responses peaking on day 7 differed from those peaking on day 14. Separate analysis of expression of PD-1 on influenza virus-specific CD4 T cells from young (black symbols) versus aged (red symbols) subjects was performed on day 7 post-TIV (Mann-Whitney) (**G**).

Influenza virus vaccines are less effective in the aged, at least in part due to a poor ability to generate NAb. It is currently unclear how vaccination with TIV impacts virus-specific CD4 T cell responses in aged individuals. We therefore examined boosting of influenza virus-specific CD4 T cells in younger and aged subjects following TIV. Virus-specific CD4 T cell responses were detected between days 7 and 14 with most responses returning to baseline levels by day 60 post vaccination. Responses of most individuals also peaked between days 7 and 14 with a few peaking later at day 28 (data not shown). However, the day of the peak of CD4 T cell responses varied from individual to individual (Figure **2A and B**). Representative responses shown in Figure **2B** illustrate increased percentages of CD4 T cells responding on day 7 and day 14 with a marked decrease in percent of responding cells on day 10 post-vaccination in most subjects. We observed no differences in the peak magnitude of the influenza virus-specific CD4 T cell response between young and aged subjects (Figure **1C**), and the magnitude of the responses on day 7 and 14 did not differ within an age group (Figure **2C**). Interestingly, while more young subjects demonstrated responses peaking on day 7 and day 14, in the aged cohort fewer subjects had responses to H1 and H3 that peaked on day 14 (Figure **2D**). Put another way, similar numbers of younger subjects had CD4 T cell responses peaking on day 7 and 14, whereas more aged individuals had peak responses on day 7 compared to day 14. These observations support a possible deficiency of aged individuals to maintain, or mount a later influenza vaccine-specific CD4 T cell response compared to younger subjects. Recent data have demonstrated that cytokine production by influenza virus-specific CD4 T cells may be altered based on how many times these cells have been stimulated. CD4 T cells responding to novel epitopes of more recently circulating virus strains retained a Th0 phenotype and produced more IL-2 and less IFNγ while cells responding to conserved epitopes and presumably stimulated repeatedly due to a history of vaccination and/or infection, produced less IL-2 and more IFNγ [24]. In agreement with these recent studies, we observed an increase in H1 and NP+M responding CD4 T cells producing IL-2 in young TIV responders compared to the aged group (Figure **2E**). These data suggest that younger subjects may be able to mount more vigorous responses to novel epitopes of new influenza virus strains compared to aged individuals, creating a more robust vaccine-induced response. There were no clear phenotypic differences in expression of CD27, CD45RA or PD-1 by CD4 T cells on day 7 versus day 14 when responses of young and aged subjects were combined suggesting that the day post vaccination did not influence the activation state of the CD4 T cells analyzed (Figure **2F**). However, vaccine responding CD4 T cells from aged individuals expressed higher PD-1 on day 7 compared to responding CD4 T cells from the young (Figure **2G and S4**). Thus, a shortened TIV-specific CD4 T cell response and increased expression of PD-1 on day 7 in aged subjects suggest a substantially altered boost of influenza-specific memory CD4 T cell in the aged compared to young subjects with potential implications for protective immunity. 

### Pre-existing NP and M-specific responses are robustly boosted by TIV

**Figure 3:**
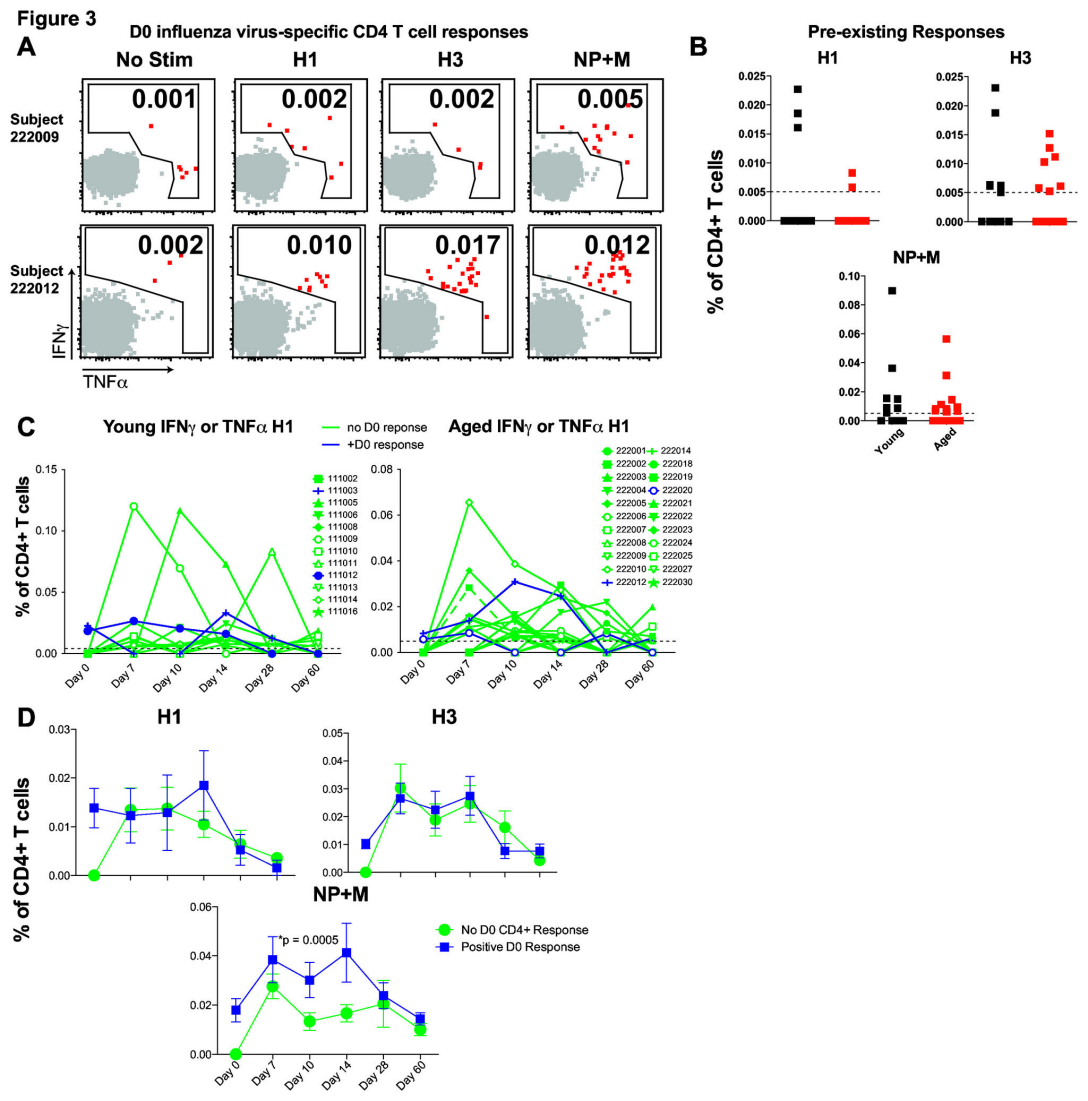
Influence of pre-existing influenza virus-specific CD4 T cells on TIV response. Representative flow cytometry plots of subjects without (top) or with (bottom) significant day 0 positive responses are shown (percent of responding CD4 T cells is indicated) (**A**). Pooled data summarize day 0 influenza virus-specific CD4 T cells for each viral protein in young (black) or aged (red) subjects (**B**). Dotted line indicated the limit of detection based on background (no stim control). Graphs of H1 responses over time after TIV in young (left) or aged (right) subjects with no day 0 influenza virus-specific CD4 T cells (green) or positive day 0 responses (blue) (limit of detection marked by dotted line) (**C**). Summary data with mean and standard error of CD4 TIV response in combined young and aged subjects with no day 0 influenza virus-specific CD4 T cells (green) or positive day 0 responses (blue) for each influenza virus protein (ANOVA) (**D**).

The impact of yearly influenza vaccination or repetitive influenza virus infections on memory CD4 T cell responses is not well defined. We hypothesized that responses against TIV might differ based on the presence or absence of influenza virus-specific memory CD4 T cells from previous infection or vaccinations and that such pre-existing immunity might differ between aged and young individuals. To test these ideas, we investigated whether the ability to mount a vaccine-induced CD4 T cell response was related to the presence of influenza virus-specific CD4 T cells prior to vaccination. There were no obvious differences in the frequency of pre-existing influenza virus HA- or NP+M-specific CD4 T cell responses between the young and aged cohorts at day 0 (Figure **3B and C**, data not shown)[16]. To test whether the presence of memory CD4 T cells prior to vaccination impacts the magnitude of the post vaccine CD4 T cell response, we examined subjects with or without robust pre-existing influenza-specific CD4 T cells (Figures **2B**, **3A and B**). For these analyses we examined young and aged subjects together to obtain enough subjects in the positive and negative pre-existing CD4 T cell response groups. Although subsequent CD4 T cell responses to H1 and H3 did not differ in subjects with or without pre-existing HA responses (Figure **3C and D**), responses to the NP+M peptides were increased in subjects with pre-existing responses against NP+M (Figure **3D**). Not only do these data indicate that CD4 T cells differ in their response to vaccination based on which viral protein they respond to (HA versus NP+M), but these results also suggest that TIV efficiently boosts pre-existing responses to NP+M, compared to HA proteins. 

### Pre-existing influenza virus-specific NAb are associated with weaker vaccine-induced CD4 T cell responses

**Figure 4:**
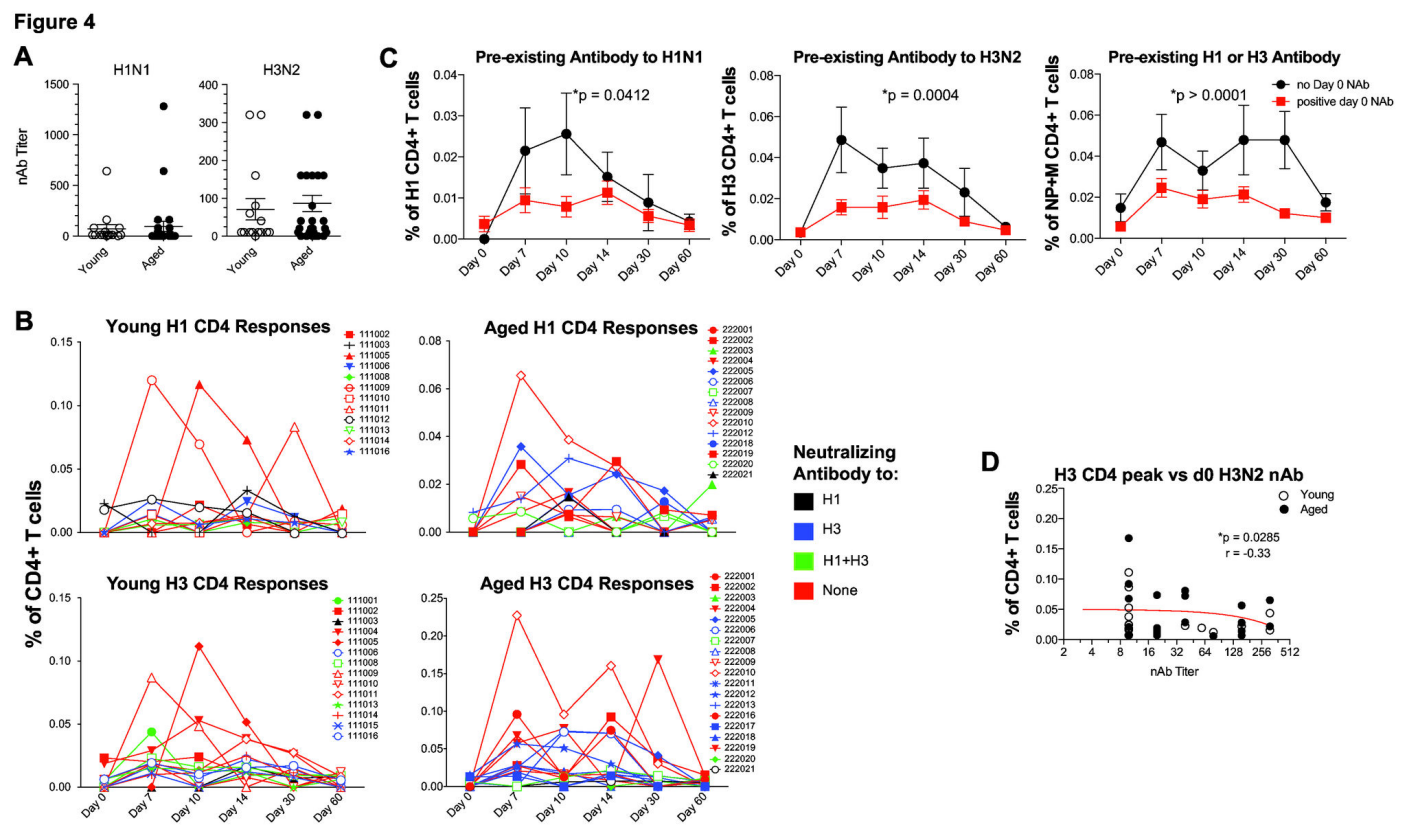
Effect of pre-existing NAb on TIV CD4 responses. Day 0 NAb titers are shown for H1N1 (left) or H3N2 (right) in young (open symbols) or aged (closed symbols) subjects (**A**). Kinetics of CD4 T cell responses in young (left) or aged (right) to H1 (top) or H3 (bottom) in relation to presence or absence of day 0 NAb to H1 (black), H3 (blue) both H1 and H3 (green), or neither strain (red) (**B**). Magnitude of CD4 T cell responses combining data for young and aged subjects to H1 (left), H3 (center), or NP+M (right) for subjects with (red) or without (black) day 0 NAb titers (ANOVA) (**C**). Correlation of NAb to H3N2 (x axis) versus peak anti-influenza virus H3 CD4 T cell response (y axis) is shown for all subjects (young - open circles; aged - closed circles) (**D**).

Although CD4 T cells are important for generating NAb, there is some debate as to whether CD4 T cells and antibodies correlate following influenza vaccination [10,28,29]. Moreover, the role of pre-existing Ab in vaccine-induced CD4 T cell expansion is not well defined. To examine this question, we compared increases in TIV-induced CD4 T cell responses between younger and aged subjects with different levels of pre-existing NAb. Both younger and aged subjects were able to generate an antibody response to influenza virus vaccination (**data not shown**), regardless of whether NAb were present prior to vaccination. However, we were interested in whether pre-existing NAb would change the CD4 T cell response to TIV. We observed no overall differences in the levels of pre-existing NAb in aged and young individuals though there was a range of NAb titers within these cohorts (Figure **4A**). There were also no obvious differences in the kinetics of influenza virus-specific CD4 T cells based on the presence of pre-existing neutralizing antibodies, and boosting of CD4 T cell responses was observed in subjects who had pre-existing antibodies to H1N1, H3N2 or both viral subtypes (Figure **4B**). When young and aged subjects were combined for analysis there were, however, significant differences in the magnitude of CD4 T cell responses when comparing subjects with or without influenza virus NAb prior to TIV (Figure **4C**). Subjects with day 0 influenza-specific NAb titers had decreased CD4 T cell responses to H1, H3 and NP+M viral proteins at multiple time points after vaccination. Indeed a negative correlation was found between the titer of pre-existing H3 neutralizing antibodies and the peak magnitude of CD4 T cell responses to influenza virus H3 (Figure **4D**). These data suggest the possibility that NAb may neutralize antigen and/or prevent optimal antigen presentation to T cells. More importantly these data suggest that yearly vaccination inducing NAb to influenza virus may impede robust boosting of virus-specific CD4 T cell responses.

## Discussion

Influenza virus infection is an important public health concern as existing vaccines are suboptimal, especially in the elderly [1]. Here, we examined how the current TIV boosts virus-specific CD4 T cell responses in younger and aged humans. We identified several important features of CD4 T cell stimulation by TIV. First, TIV induced 2 peaks of CD4 T cell responses on days 7 and 14 post vaccination. Aged subjects had a prominent bias towards peak responses on day 7 while in younger subjects responses peaked on both days 7 and 14. Second, TIV robustly boosted NP+M specific CD4 T cells and pre-existing responses to NP+M correlated with enhanced boosting of these responses. Interestingly, pre-existing HA-specific CD4 T cells had little effect on the magnitude of the HA-specific CD4 T cell boost. Third, pre-existing NAb correlated with a reduced vaccine-induced CD4 T cell boost. Given the recently appreciated potential of Th1-like memory CD4 T cells to contribute to protective immunity to influenza virus [5,7], these observations may have important implications for optimizing influenza virus vaccine approaches, especially in the elderly. 

The identification of 2 peaks of vaccine induced CD4 T cells was somewhat surprising and is unusual for vaccine-induced T cell responses in humans [30]. However, mouse models have shown concurrent primary and secondary T cell responses [31] as well as differences between primary and secondary response kinetics [32], and protective capacity [12]. Of course, for influenza virus-specific CD4 T cells identified with the approaches used here, we cannot distinguish whether responses on days 7 and 14 represent T cells targeting the same epitopes or whether there are sequential waves of T cells responding to different determinants. One possibility is that the responses observed on day 14 represent 1° or previously weakly stimulated T cells while the responses on day 7 post vaccination represent cells that have previously been primed and/or boosted (2°, 3°, etc responses). Such an interpretation would be consistent with the notion that aged humans have accumulated a lifetime of exposures to influenza virus through infections and vaccinations resulting in a higher proportion of repetitively stimulated T cells. However, it is unclear in such a model why the magnitude of these responses is not larger suggesting that an upper limit to the size of these responses may exist. It should be noted that the magnitude of the vaccine induced CD4 T cell responses observed here (0-0.227% of CD4 T cells) are in good agreement with previous studies using ELISPOT or ICS following peptide pool or whole virus stimulation [7,8,24], suggesting that TIV induced CD4 T cell responses are typically in the 0.005-0.1% range, at least for Th1-like responses. An alternative possibility is that boosted CD4 T cells in aged subjects may undergo more rapid contraction. This possibility seems unlikely as naïve CD4 T cells in aged mice are longer lived [18], a property associated with decreased Bim expression in mice [19], a change we have also observed in humans (Figure **S5**). However, an increase in senescent T cells in the aged [8,18] could lead to decreased survival following boost. A third possibility is a decrease in repertoire diversity in the aged leading to fewer new (i.e. 1°) T cell responses upon vaccination [24]. Although, we observed no negative correlation between the size of the naïve CD4 T cell pool and the magnitude of the CD4 T cell response (data not shown), this study was not powered to detect small changes and there is previous evidence of constriction of the naïve T cell repertoire with age [33,34]. In the future it will be interesting to analyze how differences in naïve CD4 T cells correlate with TIV-induced responses in aged subjects. 

NP+M-specific CD4 T cell responses were significantly boosted by TIV administration. In contrast, pre-existing CD4 T cells specific for HA or NA appeared to have little correlation with post-vaccination expansion of these responses. Several factors may account for this difference in CD4 T cell responses. Variable amounts of each viral protein have been detected in TIV preparations [27,35]. Although HA protein in the vaccine is quantified to contain 15μg of HA protein for each dose, the relative amount of other proteins may differ [36]. Although one TIV prep has been reported to contain 22μg of NP [37], this has not been extensively evaluated for different TIV lots. However, specific epitopes that are conserved among TIV vaccines and circulating viruses from year to year may also influence boosting of CD4 T cell responses. Recent data suggest repetitive priming with influenza virus antigen induces Th1 CD4 T cells, while recently primed CD4 T cells to novel influenza virus epitopes may adopt a more Th0 phenotype lacking IFNγ production, but producing IL-2 [24]. Younger subjects may be better able to mount new Th0 responses to novel influenza virus epitopes compared to aged subjects because of a more diverse TCR repertoire. In addition aged individuals may accumulate a broader diversity of influenza virus-specific memory Th1-like CD4 T cells leading to a type of “original antigenic sin” where predominantly pre-existing responses are stimulated by vaccination. The NP protein in the 2012-2013 vaccine strain (A/California/7/2009) has 94% identity with the 1918 H1N1 (A/Brevig Mission/1/1918) strain and nearly 90% identity to vaccine strains from the past 20 years. The H1 protein identity is closer to 80% conserved for the last two decades suggesting that protein sequence conservation could contribute to these effects. Indeed, vaccines based on NP and/or M have been proposed to take advantage of this conservation [3,4,38]. Development of a vaccine based on enhancing NP+M responses would be a logical extension of these data, though it remains unclear whether the protein target of CD4 T cells impacts their protective capacity or whether memory Th1-like CD4 T cells are, in fact, protective in aged humans. Moreover, it is unclear if NP+M specific CD4 T cells could provide help for generating neutralizing antibodies against HA. Systematic quantification of NP and M in current vaccines and how it is related to vaccine efficacy would be of considerable interest.

The goal of TIV vaccination is to elicit protective NAb responses. However, recent studies in mice and humans have highlighted the important role of T cells and potential cooperativity between T cells and antibody in protection [7,28,39-42]. In the current study, we found that individuals with pre-existing NAb to influenza virus had decreased CD4 T cell boosting following TIV vaccination. Pre-existing NAb to H1 or H3 also appeared to blunt boosting of CD4 T cell responses to NP+M. This observation may reflect antigen clearance by antibody preventing boosting of T cells. For example, a correlation between NAb and NP+M binding antibody might lead to a scenario where NP+M antibody simply clears this protein preventing efficient processing and presentation to CD4 T cells. It is unclear, however, whether NP- or M-specific antibodies, which would not neutralize influenza virions, can directly clear NP or M antigen in the split TIV. We saw no correlation between overall influenza virus-binding Ab and either NAb or CD4 T cell responses, though the interpretation of total binding antibody may be complex. It is also possible that the NAb itself directly clears antigen complexes of HA and NP+M leading to an HA-mediated clearance of NP and M proteins. While the precise mechanism for this effect is unclear, lack of correlation between T cell responses and NAb has been shown in aged compared to young subjects [10,29] and may be caused by increased interference of Ab with CD4 T cell access to antigen. Along with T cell and antibody responses being impaired in elderly, this lack of concordance may reflect more striking dysfunction in immune responses with age.

Effective immunization is critical for protection of aged individuals from significant morbidity or mortality due to influenza virus infection. Although TIV may be effective in healthy young individuals, differences in the vaccine-induced CD4 T cell response in aged individuals may provide clues to help optimize influenza immunity in this vulnerable population. Our data now suggest an important relationship between pre-existing NAb and NP+M-specific memory CD4 T cells and the ability of TIV to boost cellular immunity. While memory CD4 T cells specific for conserved determinants have been implicated in protection in animal models and young humans, dissecting the precise role of this arm of influenza immunity in aged humans is an important goal for future studies.

## Supporting Information

Figure S1 **We determined our response cutoff by rounding up from the 95% confi- dence interval of our negative control (0.0044% of CD4+ T cells)(mean and 95% CI shown in red).**
(EPS)

Figure S2 **Examples of young (A) and aged (B) subjects showing gating strategy from toal PBMCs, singlets,** Live and Dump, CD3, and CD4 gates. No stimulation controls used to set cytokine gates and H3-specific responses are shown for five young and five aged subjects. Numbers show in each plot represent the percentage of the parent population for the shown gate(s). H3 cytokine production is shown as raw value, from which the background no stimulation control data would be subtracted for all subsequent statistical analysis. 
(EPS)

Figure S3 **PD-1 MFI in day 0 (closed symbols) and day 7 (open symbols) CD4 T cells responding to H1, H3, NP+M, or SEF (Mann-Whitney).**
(EPS)

Figure S4 **PD-1 expression on day 7 responding CD4 T cells to H1, H3, or NP+M from young (black) or aged (red) subjects (ANOVA). **
(EPS)

Figure S5 **Bim MFI in total CD4 T cell from young (closed symbols) and aged (open symbols) CD4 T cells.** Red lines show mean and SEM (Mann- Whitney).
(EPS)
